# Interplay of Temperature-Induced Modification in Niobium Oxide Thin Films for Electrochromic Advancements

**DOI:** 10.3390/ma18061264

**Published:** 2025-03-13

**Authors:** Rutuja U. Amate, Pritam J. Morankar, Namita A. Ahir, Chan-Wook Jeon

**Affiliations:** School of Chemical Engineering, Yeungnam University, 280 Daehak-ro, Gyeongsan 712-749, Republic of Korea; rutu.nanoworld@gmail.com (R.U.A.); pritam.nanoworld@gmail.com (P.J.M.); namita.nanotechnology@gmail.com (N.A.A.)

**Keywords:** electrochromism, niobium oxide, hydrothermal, temperature-dependent, optical modulation

## Abstract

Niobium oxide (Nb_2_O_5_) is a compelling preference for electrochromic (EC) applications due to its remarkable optical modulation, chemical resilience, and efficient charge accommodation. This study attentively explores the influence of reaction temperature on the structural, morphological, and EC characteristics of Nb_2_O_5_ thin films synthesized via a hydrothermal approach. Reaction temperatures spanning 140 °C to 200 °C were optimized to unravel their pivotal role in dictating material properties and device performance. Field-emission scanning electron microscopy elucidates significant morphological transformations, transitioning from agglomerated, cracked structures at lower temperatures to well-defined, porous architectures at optimal conditions, followed by a re-compaction of the surface at elevated temperatures. Electrochemical analysis established a strong correlation between thermal-induced structural refinements and enhanced EC performance metrics. The optimized N-180 thin films exhibit enhanced charge injection dynamics, improved coloration efficiency of 81.33 cm^2^/C, and superior optical modulation of 74.13% at 600 nm. The device fabricated with the most favorable film demonstrated significant optical contrast and long-term stability, reinforcing its practical viability for smart window and energy-efficient applications. This study pioneers a comprehensive understanding of the thermal modulation of Nb_2_O_5_ thin films, providing new insights into the interplay between reaction temperature and material functionality.

## 1. Introduction

In the modern technological era, energy-efficient materials have gained paramount importance due to the increasing demand for sustainable and intelligent systems. Among these, electrochromic (EC) materials have emerged as a key class of materials capable of dynamically modulating their optical properties triggered by redox reactions in response to an applied voltage [1]. These materials are widely explored for applications such as smart windows, energy-saving displays, rear-view mirrors, and advanced optoelectronic systems [2,3]. Smart windows, which dynamically control light transmission to reduce energy wastage in buildings, represent a key area where electrochromic materials can significantly contribute to sustainability. Among various EC materials, transition metal oxides (TMOs) such as tungsten oxide (WO_3_), titanium oxide (TiO_2_), molybdenum oxide (MoO_3_), nickel oxide (NiO), manganese dioxide (MnO_2_), and niobium oxide (Nb_2_O_5_) have garnered significant attention due to their reversible EC behavior, multiple redox states, and robust structural stability [4,5,6]. In particular, niobium oxide (Nb_2_O_5_), a wide-bandgap TMO, has emerged as a promising EC material owing to its superior chemical stability, excellent charge storage properties, high optical transparency in the visible and near-infrared regions, and exceptional corrosion resistance in acidic and basic electrolytes [7,8]. Unlike traditional EC materials such as tungsten oxide (WO_3_) that exhibit strong coloration but suffer from cycling degradation, Nb_2_O_5_ offers prolonged EC durability while maintaining adequate optical modulation. Additionally, the ability of Nb_2_O_5_ to facilitate intercalation/deintercalation of small cations (Li^+^) with minimal structural distortion makes it an ideal host material for electrochromic and energy storage applications. Its capability to reversibly insert and extract charge carriers has positioned it as a suitable candidate for multifunctional applications such as electrochromic supercapacitors, energy storage-integrated smart windows, and optoelectronic systems [3,9]. The EC performance of Nb_2_O_5_ is highly dependent on its structural and morphological characteristics, which can be tuned via synthesis conditions, precursor selection, and deposition techniques. Among the various synthesis methods available for fabricating Nb_2_O_5_ thin films, hydrothermal synthesis has proven to be an effective and scalable approach, offering superior control over crystallinity, phase purity, and nanostructural features [10]. However, the EC response of Nb_2_O_5_ is intrinsically linked to its phase evolution and crystallographic modifications, which are strongly influenced by the synthesis temperature. The structural phase transition of Nb_2_O_5_ is a crucial determinant of its electrochemical and optical properties, as different polymorphic phases exhibit distinct EC characteristics. Typically, the formation of the orthorhombic T-Nb_2_O_5_ phase is considered optimal for EC applications due to its favorable Li^+^ ion insertion/extraction kinetics, high coloration efficiency, and enhanced cycling stability [11].

Despite its advantages, the widespread adoption of Nb_2_O_5_ for EC applications is hindered by challenges such as relatively low coloration efficiency, and slow switching kinetics [12]. These limitations are often linked to the material’s crystallinity, porosity, and ion diffusion pathways, which can be systematically controlled through precise synthesis techniques. Hydrothermal synthesis, in particular, offers an efficient and scalable route for fabricating high-purity Nb_2_O_5_ thin films with tunable nanostructures and enhanced EC performance. An extensive studies on Nb_2_O_5_ thin films, the systematic investigation of temperature-driven modifications in its structural, morphological, and EC properties remains an area of interest. Temperature variations during the hydrothermal synthesis process significantly impact the crystallinity, phase composition, and surface morphology of Nb_2_O_5_ films, which in turn modulate their EC switching dynamics, optical modulation, and charge storage behavior [13]. A comprehensive understanding of these temperature-induced modifications is essential to optimize the deposition conditions for high-performance EC devices.

In this study, we investigate the interplay between synthesis temperature and electrochromic properties in Nb_2_O_5_ thin films fabricated via a hydrothermal route. A series of Nb_2_O_5_ thin films were synthesized at varying temperatures to elucidate their structural evolution, phase transformation, and EC performance. The optimized Nb_2_O_5_ film synthesized at 180 °C exhibits a well-defined structure, which contributes to its superior EC response. Detailed structural, morphological, and electrochemical analyses were performed to correlate temperature-dependent modifications with EC kinetics. This work not only advances the knowledge of temperature-induced phase transformations in Nb_2_O_5_ but also lays the foundation for optimizing deposition conditions to achieve high-performance EC materials for next-generation smart windows and energy-efficient devices. The integration of structural engineering and EC functionality in Nb_2_O_5_ thin films underscores their potential for multifunctional applications, further bridging the gap between energy-efficient technologies and sustainable material development.

## 2. Experimental Section

### 2.1. Reagents and Materials

All reagents used for the synthesis were of analytical grade and required no additional purification. Fluorine-doped tin oxide (FTO) glass substrates (MTI. Co., Ltd., Seoul, South Korea) were employed as the conductive support for Nb_2_O_5_ thin films. The substrates were pre-cleaned through sequential ultrasonication in ethanol, acetone, and deionized (DI) water for 15 min each. Niobium chloride (NbCl_5_, Sigma–Aldrich, 99%, St. Louis, MO, USA), oxalic acid (C_2_H_2_O_4_, Alfa Aesar, 98%, Haverhill, MA, USA), lithium perchlorate (LiClO_4_, Sigma–Aldrich), and propylene carbonate (PC, Sigma–Aldrich) were used as received. DI water was used as the solvent throughout the synthesis process.

### 2.2. Synthesis of Nb_2_O_5_ Thin Films

**Figure 1:**
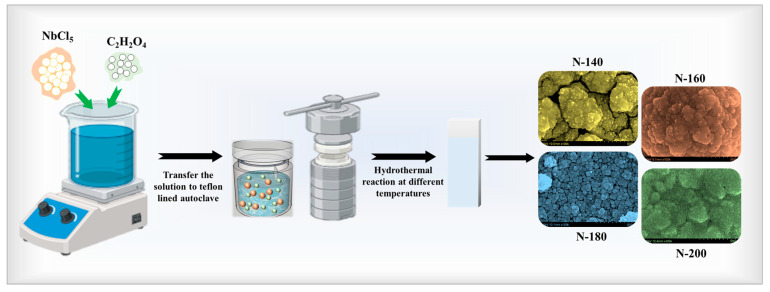
Schematic illustration of the synthesis process for Nb_2_O_5_ thin films via the hydrothermal method.

Nb_2_O_5_ thin films were synthesized on FTO glass substrates using a hydrothermal method to investigate the influence of reaction temperature on their structural and EC properties. Initially, a precursor solution was prepared by dissolving 0.05 M NbCl_5_ and 0.1 M oxalic acid in 20 mL DI water. The mixture was stirred continuously for 10 min until a homogeneous and transparent solution was obtained. The precursor solutions were freshly prepared before each hydrothermal reaction and used after 10 min of stirring to ensure consistent film formation. The prepared solution was then transferred into a 50 mL Teflon-lined stainless-steel autoclave. Pre-cleaned FTO glass substrates were placed vertically inside the autoclave to ensure uniform film deposition on their surfaces. The autoclave was sealed and subjected to hydrothermal reactions at four different temperatures, 140 °C, 160 °C, 180 °C, and 200 °C, for a duration of 6 hours, at a controlled rate of 5 °C/min to ensure uniform growth of Nb_2_O_5_ thin films. This heating rate prevents sudden temperature gradients, which can lead to heterogeneous film formation. The hydrothermal temperature plays a critical role in determining the morphology and growth rate of thin films. After completion of the hydrothermal process, the autoclave was allowed to cool naturally to room temperature. The resultant Nb_2_O_5_ thin films were carefully removed, rinsed thoroughly with DI water to eliminate residual precursors, and annealed in a muffle furnace at 600 °C for 2 h. The annealing process was carried out in ambient air, as oxygen plays a crucial role in stabilizing the desired T-Nb_2_O_5_ orthorhombic phase. The synthesized films were labeled based on their hydrothermal reaction temperatures, N-140, N-160, N-180, and N-200, for subsequent characterization and EC analysis. The potential representation of the synthesis process for Nb_2_O_5_ thin films is presented in Figure 1.

### 2.3. Electrochromic Device Fabrication

An electrochromic (EC) device was fabricated using the N-180 thin film synthesized at the optimized reaction temperature. The device had a Glass/FTO/N-180/LiClO_4_ + PC/FTO/Glass configuration. The FTO-coated glass served as the conductive electrodes, while the N-180 thin film acted as the EC active layer. A 1 M solution of lithium perchlorate dissolved in propylene carbonate was used as the electrolyte. The electrolyte was sandwiched between the active EC layer and the counter electrode, with the assembly being secured using transparent, adhesive, double-sided tape (Scotch Brand Tape, 3M, St. Paul, MN, USA). The fabricated device was sealed to prevent electrolyte leakage and ensure long-term stability.

## 3. Sample Characterization and Electrochemical Measurements

The structural, morphological, optical, and electrochemical properties of the Nb_2_O_5_ thin films synthesized at varying hydrothermal temperatures were characterized using X-ray diffraction (XRD; PAN Analytical, Almelo, The Netherlands), field-emission scanning electron microscopy (FE-SEM, S-4800 HITACHI, Ltd., Tokyo, Japan) complemented by (energy dispersive spectroscopy, (EDS)), and X-ray photoelectron spectroscopy (XPS; K-Alpha, Thermo Scientific, Cheshire, UK).

Electrochemical analyses were performed using a three-electrode system integrated with a battery cycler (Biologic Instrument-WBCS3000, Gières, France). The EC performance, specifically the optical transmittance, was evaluated using a UV-Vis spectrophotometer (Model: S-3100, SCINCO, Seoul, South Korea) in conjunction with an electrochemical workstation (IVIUM Technologies, COMPACTSTAT.e, Capelle aan den IJssel, The Netherlands). All measurements were carried out in a 1 M LiClO_4_+PC electrolyte solution. The Nb_2_O_5_ films, synthesized under varying reaction temperatures, served as the working electrode, while a platinum electrode and an Ag/AgCl electrode were employed as the counter and reference electrodes, respectively. This configuration facilitated a comprehensive investigation of the electrochemical and optical properties of the films under operational conditions.

## 4. Results and Discussion

### 4.1. Structural and Compositional Characteristics

**Figure 2:**
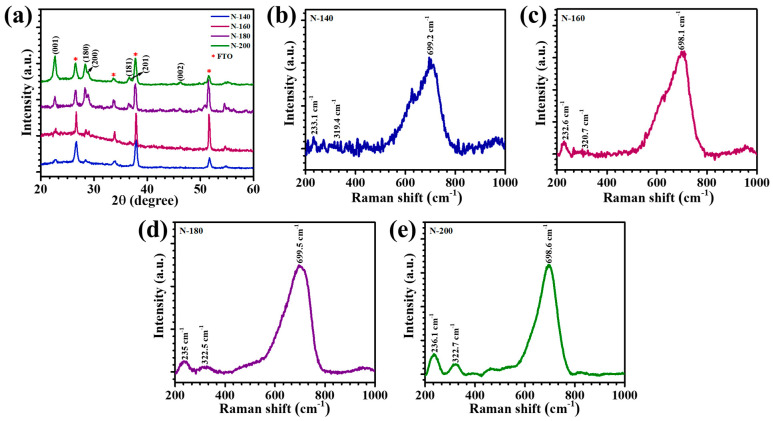
(**a**) XRD patterns of Nb_2_O_5_ thin films synthesized at different hydrothermal temperatures, (**b**–**e**) Raman spectra of N-140, N-160, N-180, and N-200 thin films.

The XRD patterns of Nb_2_O_5_ thin films synthesized at varying hydrothermal temperatures (140 °C, 160 °C, 180 °C, and 200 °C) were analyzed to elucidate their crystalline structure, phase purity, and temperature-induced structural variations, presented in Figure 2a. The XRD patterns exhibit sharp and well-defined peaks, underscoring the crystalline nature of the samples. The stacked XRD profiles of samples N-140, N-160, N-180, and N-200 were compared to assess the influence of hydrothermal temperature on crystallinity and structural evolution. All films displayed peak orientations consistent with the orthorhombic phase of Nb_2_O_5_ (T-Nb_2_O_5_), as confirmed by their alignment with JCPDS card no. 00-030-0873. Prominent diffraction peaks were observed at 22.60°, 28.40°, 28.87°, 36.99°, and 46.15°, 2θ, corresponding to the (001), (180), (200), (181), (201), and (002) crystallographic planes, respectively. Among these, the diffraction peaks at 22.60° and 28.40° exhibited higher intensities, suggesting a preferential orientation of the crystal structure along the (001) and (180) planes. Such a dominant orientation may enhance electrochemical interactions, particularly with Li^+^ ions, which are crucial for EC applications [3]. The impact of hydrothermal temperature on crystallinity was evident in the relative peak intensities across the samples. The N-200 and N-180 films demonstrated significantly sharper and more intense diffraction peaks compared to N-140 and N-160, indicating enhanced crystallinity at higher reaction temperatures. This can be attributed to the improved nucleation and growth kinetics of Nb_2_O_5_ crystals under elevated thermal conditions [14]. Conversely, the films synthesized at 140 °C and 160 °C exhibited comparatively broader peaks with lower intensities, signifying a less developed crystalline framework. Importantly, no secondary or impurity phases were detected in the XRD patterns, confirming the phase purity of all samples.

Raman spectroscopy was utilized to investigate the structural and vibrational properties of Nb_2_O_5_ thin films synthesized at varying hydrothermal temperatures: 140 °C (N-140), 160 °C (N-160), 180 °C (N-180), and 200 °C (N-200). The Raman spectra, depicted in Figure 2b–e, provide valuable insights into the crystallinity, chemical bonding, and vibrational modes of the Nb_2_O_5_ thin films. The characteristic orthorhombic T-Nb_2_O_5_ phase, achieved after annealing at 600 °C, is defined by distorted octahedral or pentagonal bipyramid units where each Nb atom is coordinated with six or seven oxygen atoms. These polyhedral units are connected by edge- or corner-sharing along the *ab* plane and by corner-sharing along the *c* axis [15]. This intricate structural arrangement was evident in the Raman spectra of all the samples, confirming the successful formation of the T-Nb_2_O_5_ phase. The dominant Raman peak observed in the ~698–699 cm^−1^ range across all samples is attributed to the symmetric and asymmetric stretching vibrations of Nb–O bonds within the octahedral and polyhedral frameworks. This peak is an assertion of the orthorhombic T-Nb_2_O_5_ phase and highlights the structural stability of the films across the varying synthesis temperatures. Additional peaks located at ~319–322 cm^−1^ and ~232–236 cm^−1^ correspond to the bending vibrations of Nb–O–Nb bridges and the presence of terminal Nb=O bonds, respectively [16,17]. A detailed comparison of the Raman spectra for N-140, N-160, N-180, and N-200 reveals consistent peak positions and intensities, suggesting that the hydrothermal temperature variation does not significantly alter the intrinsic vibrational properties of Nb_2_O_5_. However, subtle differences in the peak sharpness and intensity, particularly for N-200, indicate enhanced crystallinity and stronger bonding interactions at higher hydrothermal temperatures. The narrower and more intense peaks observed for N-180 and N-200 samples are indicative of improved lattice ordering and a higher degree of structural regularity.

**Figure 3:**
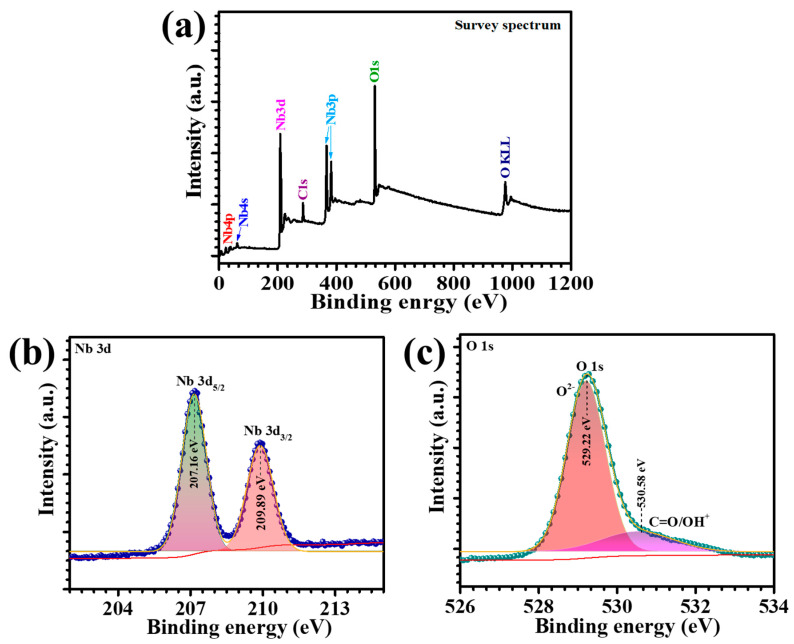
(**a**) XPS survey spectrum of the N-180 sample, high-resolution (**b**) Nb 3d spectrum, and (**c**) O 1s spectrum.

XPS was employed to investigate the stoichiometric chemical composition and the valence states of elements in the N-180 thin films. The survey scan and high-resolution core-level spectra of the N-180 Nb_2_O_5_ thin film are presented in Figure 3a–c. In the survey spectrum (Figure 3a), the predominant peaks corresponding to niobium (Nb) and oxygen (O) were clearly identified, along with a minor signal of adventitious carbon (C), which is typically attributed to surface contamination from the ambient environment. The absence of peaks for any extraneous elements underscores the high compositional purity of the hydrothermally deposited N-180 thin film. The high-resolution Nb 3d spectrum, shown in Figure 3b, revealed two distinct peaks at binding energies of 207.16 eV and 209.89 eV, corresponding to the spin-orbit doublets of Nb 3d_5/2_ and Nb 3d_3/2_, respectively, with an energy separation of 2.73 eV. These binding energy values are consistent with previously reported data for Nb in the +5 oxidation state, confirming the presence of Nb^5+^ species in the film [18]. The sharpness and well-defined nature of these peaks indicate the homogeneity and stoichiometric integrity of the Nb_2_O_5_ phase. The O 1s spectrum, as depicted in Figure 3c, displayed two individual peaks. The primary peak, centered at 529.22 eV, is attributed to oxygen ions (O^2−^) in Nb-O bonds, indicative of the Nb-O-Nb lattice in the Nb_2_O_5_ structure. A secondary shoulder peak at 530.58 eV corresponds to surface oxygen species (-OH) [19]. Notably, the area under the Nb-O-Nb peak was significantly larger than that of the -OH peak, affirming the dominance of the Nb_2_O_5_ phase within the thin film.

**Figure 4:**
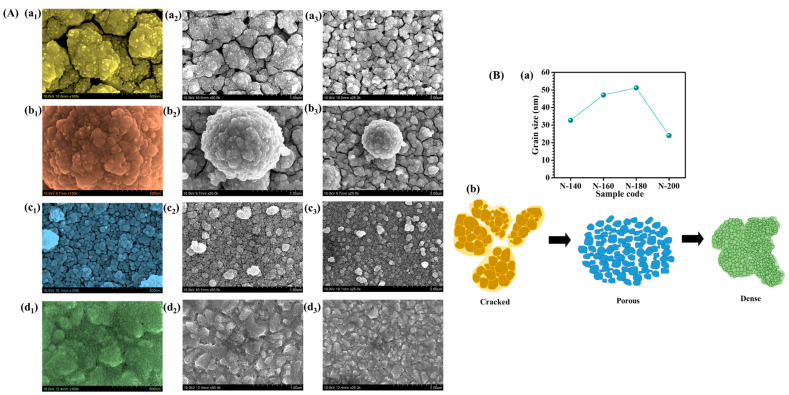
FE-SEM images of (**A**) (**a_1_**–**a_3_**) N-140, (**b_1_**–**b_3_**) N-160, (**c_1_**–**c_3_**) N-180, and (**d_1_**–**d_3_**) N-200 samples at different magnifications, (**B**) (**a**) grain size of samples, (**b**) schematic presentation of morphological evolution from cracked to porous to dense.

The FE-SEM analysis of Nb_2_O_5_ thin films synthesized at different hydrothermal temperatures (140 °C, 160 °C, 180 °C, and 200 °C) reveals a progressive transformation in surface morphology, directly influenced by the synthesis temperature. At the lower hydrothermal temperature of 140 °C (N-140), shown in Figure 4A(a_1_–a_3_), the Nb_2_O_5_ structures exhibit a highly aggregated and densely packed morphology with noticeable cracks. The limited thermal energy at this stage hinders sufficient crystal growth, leading to irregularly shaped nanoparticles with poor structural uniformity. The presence of cracks and compact regions indicates a constrained nucleation process where the particles cluster together without forming a well-defined porous network. Such a morphology may result in inefficient ion transport and hinder EC performance due to reduced electrolyte penetration. As the hydrothermal temperature increases to 160 °C (Figure 4A(b_1_–b_3_)), morphological evolution is evident, but the surface still exhibits noticeable cracks and the persistence of aggregated particle formation. While the increased thermal energy facilitates partial crystallization and structural rearrangement, the material has not yet achieved a fully developed porous network. The nano-aggregates remain densely packed and the presence of cracks suggests incomplete grain growth and structural stress within the Nb_2_O_5_ framework. This indicates that, although some degree of reorganization occurs, the nucleation process is still dominant over grain growth, limiting the formation of a well-defined porous architecture. Such morphology may lead to suboptimal ion diffusion and charge transport, making further temperature optimization essential to achieve an ideal EC performance. At 180 °C, the Nb_2_O_5_ morphology reaches an optimal state, characterized by a highly uniform porous architecture with well-defined nanospheres, depicted in Figure 4A(c_1_–c_3_). The balance between nucleation and grain growth at this temperature facilitates the development of an open-structured network, significantly enhancing electrochemical properties. The well-organized porous morphology ensures efficient electrolyte penetration, faster ion diffusion, and improved EC switching kinetics. The enhanced surface area at this stage makes the material highly suitable for EC applications, as it offers better charge accommodation and increased reaction sites. However, at an even higher synthesis temperature for N-200 sample, the morphology undergoes excessive densification (Figure 4A(d_1_–d_3_)). The increased thermal energy accelerates crystal growth at the expense of porosity, leading to a compact surface with closely packed grains. The initially porous architecture collapses into a more rigid and dense structure, reducing the number of active sites available for electrochemical reactions. This structural densification limits ion intercalation, ultimately compromising EC performance. Overall, these findings emphasize the importance of precise temperature control during synthesis to achieve the desired morphology for high-performance electrochromic energy storage applications.

To delve deeper, the grain sizes have measured from multiple regions of the FE-SEM images and have plotted the average results in the form of plot to (Figure 4B(a)) illustrate the size distribution at each temperature. The calculated sizes are as follows: N-140: 32.75 nm, N-160: 47.13 nm, N-180: 51.19 nm, N-200: 24.06 nm. These measurements reflect the changes in particle size and are presented in the revised manuscript. The variation in particle size observed in the Nb_2_O_5_ thin films synthesized at different hydrothermal temperatures (140 °C, 160 °C, 180 °C, and 200 °C) can be attributed to the thermal effects on crystal nucleation and growth processes during the hydrothermal synthesis. At 140 °C (N-140), the lower temperature provides insufficient energy for the material to undergo significant crystallization. As a result, the grains remain more aggregated and less defined (Figure 4B(b)) and the material exhibits cracks and a more compact structure. At 160 °C, the synthesis process is more thermally driven, promoting some degree of rearrangement. However, the particles are still relatively small (47.13 nm) due to the continuing dominance of nucleation over crystal growth. The grain size increases slightly, but the material remains in an aggregated state, with some cracks still visible. At 180 °C (N-180), it promotes the formation of larger and more uniform grains (51.19 nm). The increased thermal energy allows the particles to undergo further crystallization, resulting in a more defined and uniform porous structure (Figure 4B(b)). The higher temperature enhances the rate of diffusion and crystal growth, contributing to a larger particle size compared to the lower temperatures. At 200 °C (N-200), the particle size decreases (24.06 nm), which can be attributed to the excessive thermal energy that promotes rapid crystal growth, leading to the densification of the Nb_2_O_5_ structure. The high temperature accelerates the grain growth process at the expense of the formation of pores, resulting in a more compact, rigid structure with closely packed grains (Figure 4B(b)).

**Figure 5:**
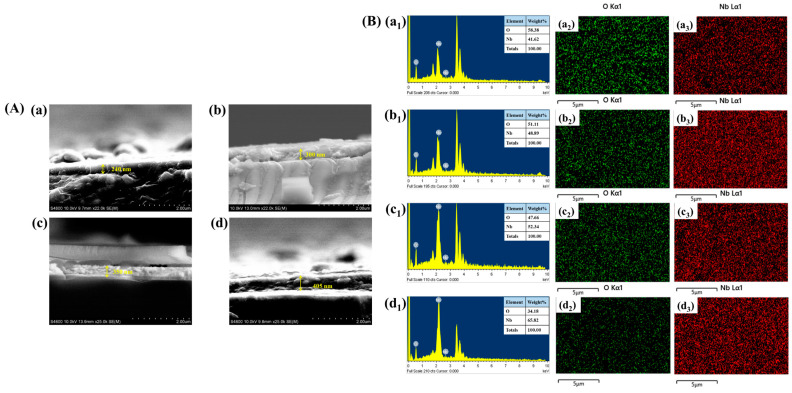
(**A**) (**a**–**d**) Cross-section images of all samples, (**B**) EDS and elemental mapping analysis of (**a_1_**–**a_3_**) N-140, (**b_1_**–**b_3_**) N-160, (**c_1_**–**c_3_**) N-180, and (**d_1_**–**d_3_**) N-200 samples.

The thickness of the Nb_2_O_5_ thin films was determined through cross-sectional analysis, shown in Figure 5A(a–d). The measured film thickness values for different reaction temperatures are 240 nm, 300 nm, 350 nm, and 405 nm for N-140, N-160, N-180, and N-200 thin films, respectively. These results indicate a clear development where the film thickness increases with higher reaction temperatures. This is attributed to enhanced nucleation and growth kinetics at elevated temperatures, leading to the formation of thicker films.

Figure 5B(a_1_–d_1_) presents the EDS spectra for the N-140, N-160, N-180, and N-200 samples, providing insights into their elemental composition. The spectra reveal the presence of Nb and O as the primary constituents, confirming the successful synthesis of Nb_2_O_5_. The inset table further quantifies the elemental composition in terms of weight percentage (wt.%), illustrating the consistency of elemental distribution across different samples. Additionally, EDS elemental mapping, as depicted in Figure 5B(a_2_–d_2_,a_3_–d_3_) for N-140, N-160, N-180, and N-200, respectively, was performed to assess the spatial distribution of Nb and O within the synthesized films. The mapping results indicate a homogeneous dispersion of elements, ensuring uniform material formation, which is crucial for maintaining consistent electrochemical and electrochromic properties.

### 4.2. Reaction Mechanism

In this study, the effect of hydrothermal temperature variation on the synthesis and reaction mechanism was meticulously explored. The synthesis begins with the dissolution of niobium chloride (NbCl_5_) in DI water, leading to the formation of hydrated niobic acid precursors as represented in Reaction (1). In the aqueous environment, the introduction of oxalic acid acts as a complexing agent, facilitating the formation of stable oxalate complexes. This step is critical as it stabilizes the Nb^5+^ ions and prevents premature precipitation. The reaction between hydrated niobic acid and oxalic acid produces [NbO(C_2_O_4_)_2_(H_2_O)_2_]^−^ and [NbO(C_2_O_4_)_3_]^3−^ complexes, as illustrated in Reaction (2). It should be noted that during the course of the reaction, the oxalate ligands may undergo decomposition or transformation. The oxalate, once coordinated to the Nb center, may either be displaced by other ligands or decompose, potentially releasing CO_2_ as a byproduct. This occurs as a result of hydrolysis or oxidative processes depending on the reaction conditions. This step is significant in understanding the overall reaction mechanism as the oxalate is no longer present in the final product, either being removed or transformed into other species, as detailed in Reaction (3). The hydrolysis of Nb^5+^ ions in solution releases OH^−^ ions, which react with the oxalate complexes to regenerate hydrated niobium oxide. The process continues with thermal dehydration at elevated hydrothermal temperatures (Δ), resulting in the formation of pure Nb_2_O_5_, as shown in Reaction (3) [16]. The hydrothermal temperature is a crucial parameter in this mechanism, as it significantly impacts the nucleation, growth kinetics, and final crystallinity of the Nb_2_O_5_ material. At lower hydrothermal temperatures, the reaction rate slows, leading to the formation of smaller or less crystalline Nb_2_O_5_ structures. Conversely, the optimum hydrothermal temperatures promote rapid dehydration and enhanced crystallinity, yielding highly ordered Nb_2_O_5_ with improved structural properties. When the hydrothermal temperature is increased beyond the optimum level, the reaction kinetics are significantly accelerated. This rapid dehydration can lead to the formation of overly dense structures with reduced porosity, potentially hindering ionic transport and active site accessibility [20].

(1) $$2{NbCl}_{5}\overset{H_{2}O}{\to }{Nb}_{2}O_{5}\cdot nH_{2}O$$

(2) $${Nb}_{2}O_{5}\cdot nH_{2}O\overset{C_{2}H_{2}O_{4}}{\to }{\left[{\left.NbO{\left(C_{2}\right.O}_{4}\right)}_{2}{\left.\left(H_{2}\right.O\right)}_{2}\right]}^{-}+{\left[{\left.NbO{\left(C_{2}\right.O}_{4}\right)}_{3}\right]}^{3-}+{C_{2}O_{4}}^{2-}$$

(3) $${\left[{\left.NbO{\left(C_{2}\right.O}_{4}\right)}_{2}{\left.\left(H_{2}\right.O\right)}_{2}\right]}^{-}+{\left[{\left.NbO{\left(C_{2}\right.O}_{4}\right)}_{3}\right]}^{3-}\overset{{2OH}^{-}}{\to }{Nb}_{2}O_{5}\cdot nH_{2}O\overset{\Delta }{\to }{Nb}_{2}O_{5}+H_{2}O$$

### 4.3. Electrochromic Characteristics

**Figure 6:**
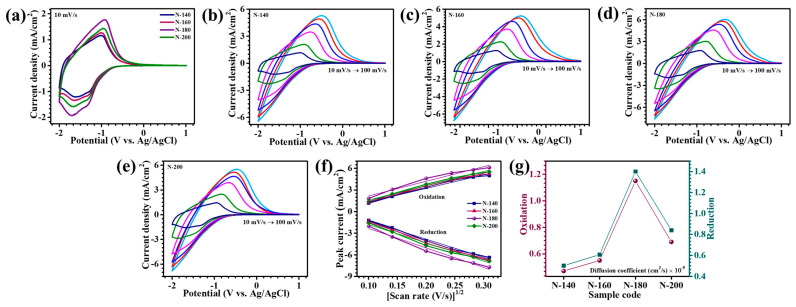
(**a**) Cyclic voltammetry profiles of all Nb_2_O_5_ thin films recorded at a scan rate of 10 mV/s, CV curves of (**b**) N-140, (**c**) N-160, (**d**) N-180, and (**e**) N-200 samples at different scan rates (10–100 mV/s), (**f**) linear relationship between peak current and square root of scan rate, and (**g**) assessment of diffusion coefficient.

The electrochemical performance of Nb_2_O_5_ thin films was systematically investigated using cyclic voltammetry (CV), chronocoulometry, and in-situ transmittance measurements in a three-electrode configuration. The electrolyte used was a 1 M LiClO_4_ + PC, enabling the intercalation and deintercalation of Li^+^ ions, a key mechanism underlying the films’ EC functionality. The CV response of Nb_2_O_5_ samples synthesized at varying hydrothermal temperatures (140 °C, 160 °C, 180 °C, and 200 °C) was recorded in the potential range of +1 V to −2 V at a scan rate of 10 mV/s (Figure 6a), allowing for a comprehensive evaluation of the effect of reaction temperature on electrochemical properties. The CV profiles revealed distinct broad redox peaks, notably at approximately −1.0 V and in the range of −1.3 V to −1.6 V, which are associated with the reversible oxidation and reduction processes facilitated by Li^+^ ion insertion and extraction within the Nb_2_O_5_ matrix. These redox reactions highlight the material’s electrochemical kinetics and EC modulation [1]. Among the samples, the Nb_2_O_5_ film synthesized at 180 °C (N-180) exhibited the most pronounced current response, indicating superior electrochemical activity. The enhanced performance of the N-180 sample can be attributed to its optimized structural and morphological characteristics, achieved through precise hydrothermal synthesis. At 180 °C, the material displayed a well-defined porous morphology and a high degree of crystallinity, which collectively facilitated efficient ion diffusion and electron transport [21]. In contrast, films synthesized at lower temperatures (N-140 and N-160) exhibited reduced electrochemical activity, likely due to the presence of structural defects, whereas the N-200 sample, fabricated at an elevated temperature, showed diminished performance due to excessive densification and a disruption in the porous network, which hindered ion accessibility and transport pathways. The CV analysis was extended across varying scan rates (10–100 mV/s) to evaluate the rate-dependent behavior and reversibility of the Nb_2_O_5_ electrodes, shown in Figure 6b–e. All samples maintained consistent CV profiles with symmetrical redox peaks across the tested range, indicating excellent structural stability and electrochemical reversibility. As the scan rate increased, a proportional broadening of the hysteresis loop was observed, reflecting enhanced Li^+^ ion insertion/extraction kinetics and higher electrochemical activity under dynamic conditions. Notably, the N-180 electrode exhibited the highest peak currents across all scan rates, highlighting its superior rate capability and capacity retention. This superior performance underscores the critical role of hydrothermal temperature in tuning the material’s structural characteristics, which directly influences ion diffusion and charge transfer efficiency.

The EC transition of Nb_2_O_5_ is governed by its reversible redox behavior, where color modulation occurs due to the intercalation and deintercalation of Li^+^ ions. During the cathodic scan, simultaneous insertion of Li^+^ ions and electrons induces the reduction of Nb^5+^ to Nb^4+^, initiating below −1 V. This reduction process leads to the formation of a deep blue coloration. On the other hand, in the anodic scan, Li^+^ ions are extracted, promoting the oxidation of Nb^4+^ to Nb^5+^. As a result, the film reverts to a transparent, bleached state, demonstrating its robust EC functionality. The redox reaction governing this process can be expressed as follows (4) [22]:

(4) $${Nb}_{2}O_{5}\left(colorless\right)+x{Li}^{+}+{xe}^{-}⇌{Li}_{x}{Nb}_{2}O_{5}\left(blue\right)$$

Figure 6f provides critical insights into the rate-determining step of the electrochemical process and the diffusion coefficient, explaining the charge-transfer kinetics. A distinct linear correlation between peak current densities (i_p_) and the square root of the scan rate (ϑ^1/2^), signifies that the Li^+^ ion insertion mechanism is predominantly governed by solid-state diffusion. To quantify the diffusion behavior, the Randles–Sevcik equation was employed (5) [23]:

(5) $$D^{\frac{1}{2}}=\frac{i_{p}}{2.69\times 10^{5}\times n^{3/2}\times A\times C\times \vartheta^{1/2}}$$

where D represents the diffusion coefficient (cm^2^/s), ip is the peak current density (mA/cm^2^), n denotes the number of electrons participating in the redox reaction (assumed to be 1), A is the working electrode’s surface area (cm^2^), C is the active electrolyte ion concentration, and ϑ signifies the scan rate (mV/s). The diffusion coefficients calculated for Nb_2_O_5_ electrodes at a 10 mV/s scan rate revealed a systematic trend. During the oxidation process, N-180 exhibited the highest diffusion coefficient (1.15 × 10^−9^ cm^2^/s), followed by N-140 (0.47 × 10^−9^ cm^2^/s), N-160 (0.55 × 10^−9^ cm^2^/s), and N-200 (0.69 × 10^−9^ cm^2^/s). Similarly, during reduction, the diffusion coefficients followed a comparable order, with N-180 attaining the maximum value (1.4 × 10^−9^ cm^2^/s), surpassing N-140 (0.502 × 10^−9^ cm^2^/s), N-160 (0.607 × 10^−9^ cm^2^/s), and N-200 (0.84 × 10^−9^ cm^2^/s). The comparable performance is presented in Figure 6g. The variation in diffusion coefficients across different samples is attributed to the structural and morphological disparities in the films. A higher diffusion coefficient suggests enhanced Li^+^ mobility within the material, which is strongly influenced by its porous nanostructure. Notably, N-180 sample’s superior diffusion kinetics can be ascribed to its well-defined porous architecture, facilitating efficient ion transport through reduced diffusion pathways and abundant active sites. The interplay between the structural attributes and diffusion characteristics highlights the crucial role of film morphology in optimizing electrochemical performance [24].

**Figure 7:**
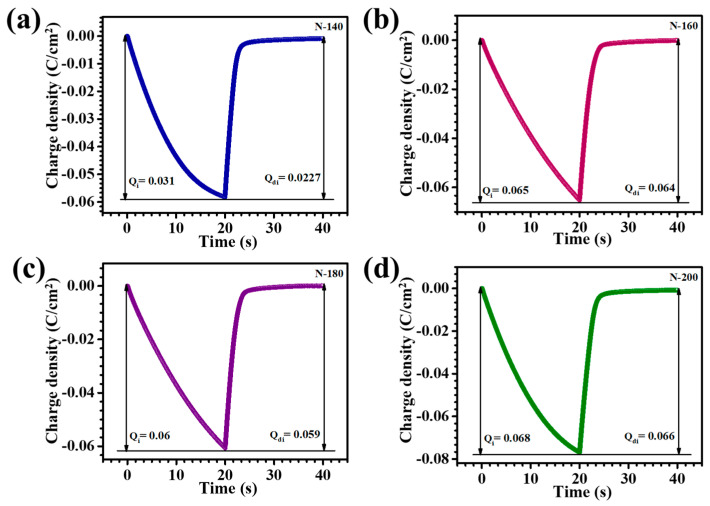
Chronocoulometry plots of (**a**) N-140, (**b**) N-160, (**c**) N-180, and (**d**) N-200 thin films.

Chronocoulometry (CC) was used to assess ion insertion and extraction during voltage application over time. The study examined how different nanostructures and reaction times affect charge dynamics, providing insights into the influence of these variables on the material’s electrochemical performance. CC measurements for the Nb_2_O_5_ electrodes were performed by applying voltage sweeps ranging from +1.0 V to −2.0 V vs. Ag/AgCl in a 1 M LiClO_4_+ PC electrolyte. This setup was used to evaluate the intercalation and deintercalation of Li^+^ ions. The charge versus time transients for the N-140, N-160, N-180, and N-200 samples are presented in Figure 7a–d. When charges are introduced into the Nb_2_O_5_ films through a diffusion process, cathodic polarization induces the reduction of Nb^5+^ to Nb^4+^, resulting in a colored state due to the intercalation of ions, typically Li^+^. During anodic polarization, the films revert to a bleached (colorless) state as the intercalated ions are extracted, with Nb^4+^ being oxidized back to Nb^5+^. This reversible process of ion intercalation (Q_i_) and de-intercalation (Q_di_) governs the EC behavior of the Nb_2_O_5_ films, allowing for the modulation of color and electronic properties based on the applied voltage. The corresponding EC reversibility of the thin films was assessed by calculating the Q_i_ and Q_di_ values, using the equation provided (6) [24]:

(6) $$Reversibility=\frac{Q_{di}}{Q_{i}}\times 100$$

Table 1 summarizes the Q_i_ and Q_di_ values along with the EC reversibility percentages for N-140, N-160, N-180, and N-200 thin films, revealing that N-180 exhibited the highest reversibility at 99%. This exceptional performance is attributed to the optimized surface area and increased density of active sites in N-180, which facilitate efficient ion insertion and extraction processes. The enhanced ion transport pathways allow for faster and more complete intercalation and de-intercalation, minimizing charge retention and improving electrochemical stability. These results emphasize the significance of precise structural optimization, particularly the role of reaction temperature in achieving superior EC performance.

**Figure 8:**
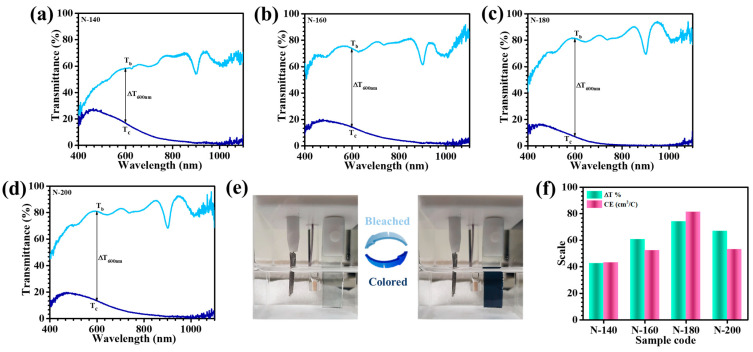
In-situ transmittance spectrum of (**a**) N-140, (**b**) N-160, (**c**) N-180, and (**d**) N-200 thin films at the colored and bleached states from 400 to 1100 nm range, and (**e**) digital photographs of N-180 thin film in both states, (**f**) comparative evaluation of electrochromic performance parameters of all samples.

The EC behavior of Nb_2_O_5_ thin films synthesized at varying hydrothermal temperatures was systematically evaluated through in-situ optical transmittance measurements. These investigations, conducted using a UV–Vis spectrophotometer coupled with an electrochemical workstation, provided profound insights into the dynamic modulation of transmittance between the colored and bleached states. Measurements covering a broad spectral range (400–1100 nm) were performed with FTO glass as the reference baseline to ensure accurate optical assessments. Figure 8a–d presents the transmittance spectra of Nb_2_O_5_ thin films fabricated at reaction temperatures of 140 °C (N-140), 160 °C (N-160), 180 °C (N-180), and 200 °C (N-200) under applied electrochemical potentials. Upon cathodic polarization at −2 V, a remarkable color transformation to deep blue was observed, which is attributed to the electrochemical reduction process involving Li^+^ insertion and charge compensation. Conversely, anodic switching to +1 V (vs. Ag/AgCl) facilitated the extraction of Li^+^ ions, restoring the films to their bleached state, thus enabling the evaluation of their optical transparency. A comparative analysis of transmittance values in both states revealed significant variations across the samples, underscoring the critical influence of hydrothermal temperature on EC performance. Table 1 consolidates the in-situ transmittance values for the bleached (T_b_ %) and colored (T_c_ %) states, along with the optical modulation (ΔT = T_b_ − T_c_) at 600 nm, a key wavelength in the visible spectrum. All samples exhibited high optical transmittance in their bleached states; however, substantial differences emerged in their coloration characteristics. Notably, the N-180 film demonstrated the lowest transmittance in the colored state (7.07% at 600 nm), dominating N-140 (16.07%), N-160 (13.41%), and N-200 (13.2%). These variations highlight the substantial impact of nanostructural and morphological attributes on EC efficiency [25]. Further supporting this, Figure 8e visually illustrates the superior color-switching capability of the N-180 film, transitioning from a fully transparent state to an intense dark blue coloration. The optical modulation parameter, which quantitatively expresses the extent of transmittance variation, strengthened these findings. The N-180 film exhibited an outstanding ΔT of 74.13% at 600 nm, significantly superior than the values observed for N-140 (42.63%), N-160 (60.64%), and N-200 (66.82%). This exceptional performance can be imputed to the optimized hydrothermal temperature, which enhances charge-transfer kinetics, promotes a highly porous and electrochemically active nanostructure, and facilitates rapid Li^+^ diffusion. Overall, the findings demonstrate that the hydrothermal reaction temperature plays a pivotal role in dictating the EC properties of Nb_2_O_5_ thin films. The N-180 sample emerged as the optimal composition, exhibiting remarkable EC reversibility, and superior optical modulation. The enhanced performance is likely a result of the film’s well-balanced nanostructure, providing both high surface area and efficient ion transport pathways, thereby maximizing electrochemical response and optical contrast [26,27].

The EC performance of Nb_2_O_5_ thin films synthesized under different hydrothermal temperatures was thoroughly assessed by evaluating their coloration efficiency (CE). As a fundamental parameter governing EC performance, CE represents the optical modulation capability of the material per unit charge density and is mathematically expressed as (7) [28]:

(7) CE=ΔODQiA

where Q_i_/A denotes the charge inserted per unit area of the working electrode, which is derived from the CC plot. The optical density variation (ΔOD) is a charge-dependent function defined by (8) [29]:

(8) $$\Delta OD=ln\frac{Tb}{Tc}$$

where T_b_ and T_c_ correspond to the transmittance values of the thin film in its bleached and colored states, respectively. This parameter fundamentally dictates the degree of optical contrast achieved during the EC switching process. Table 1 presents the ΔOD and CE values for the Nb_2_O_5_ thin films synthesized at hydrothermal reaction temperatures of 140 °C (N-140), 160 °C (N-160), 180 °C (N-180), and 200 °C (N-200). A direct correlation between ΔOD and CE was observed, with the N-180 thin film exhibiting the highest values. At 600 nm, the N-180 sample demonstrated a superior CE of 81.33 cm^2^/C, significantly incomparable to those of the other films. Figure 8f depicts the equivalent EC parameters of all samples in terms of optical modulation and CE, which can relate their performance metrics. The exceptional EC response of N-180 is intrinsically linked to its well-defined porous morphology, which provides a highly accessible framework for efficient charge transport and Li^+^ intercalation. The structural refinement at this specific reaction temperature resulted in a highly dispersed Nb_2_O_5_ matrix, enabling greater interaction between Nb atoms and Li^+^ ions. This interaction is crucial in the formation of stable niobium bronze complexes (Li_x_Nb_2_O_5_) which play a pivotal role in enhancing coloration efficiency [30]. Comparative analysis with the other samples underscores the influence of hydrothermal temperature variation on EC performance. The N-140, N-160, and N-200 films, despite exhibiting notable EC activity, displayed relatively lower ΔOD and CE values due to suboptimal structural characteristics. The N-140 sample suffered from limited ion diffusion due to its dense and less porous nature, whereas the N-200 film exhibited excessive grain growth, leading to structural coalescence that hindered effective charge intercalation. Herein, this study reveals that hydrothermal temperature plays a crucial role in determining the EC properties of Nb_2_O_5_ thin films. The optimized reaction conditions at 180 °C resulted in an ideal nanostructural arrangement, facilitating improved optical modulation, making it a strong candidate for high-performance smart window applications and other energy-efficient EC devices.

**Figure 9:**
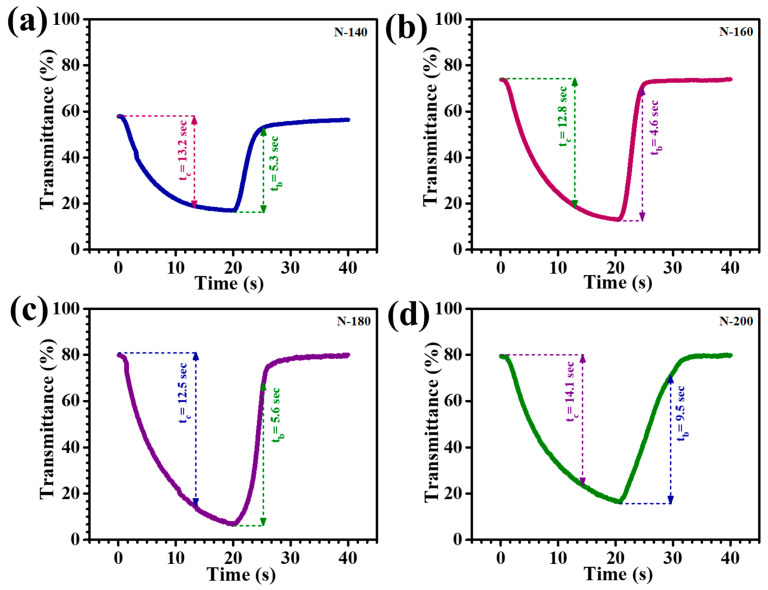
Electrochromic switching response time of (**a**) N-140, (**b**) N-160, (**c**) N-180, and (**d**) N-200 thin films in the colored and bleached state.

The rate at which EC films transition between their colored and transparent states is a critical parameter determining their functionality and practical application. The coloration time refers to the duration required for the film to attain its fully colored state, while the bleaching time indicates the time needed to revert to transparency. Rapid coloration and bleaching are essential for enhancing the usability of EC films in applications such as smart windows and electronic displays, where swift optical transitions are required to meet dynamic operational demands. In this study, the switching characteristics of Nb_2_O_5_ thin films were evaluated by monitoring the in-situ transmittance at 600 nm under a cyclic voltage step between +1 V and −2 V vs. Ag/AgCl. The switching times for coloration and bleaching were determined over a 20-s interval, providing insights into the films’ optical response and dynamic performance. The in-situ transmittance plots, shown in Figure 9a N-140, Figure 9b N-160, Figure 9c N-180, and Figure 9d N-200, illustrate the efficiency and speed of optical modulation, highlighting the potential of these heterostructures for advanced EC applications. The response time of EC films was determined as the time required to achieve 90% of the total transmittance change. These switching depend on electron transfer and the diffusion rate of Li^+^ ions. Table 1 summarizes the coloration (t_c_) and bleaching (t_b_) times for each thin film. This indicates that the Li^+^ extraction process (bleaching) is faster than insertion (coloration), consistent with typical EC behavior. The faster bleaching in N-180 thin films can be attributed to the higher electronic conductivity of the bleached states, likely due to the presence of Li_x_Nb_2_O_5_ phase [31].

**Figure 10:**
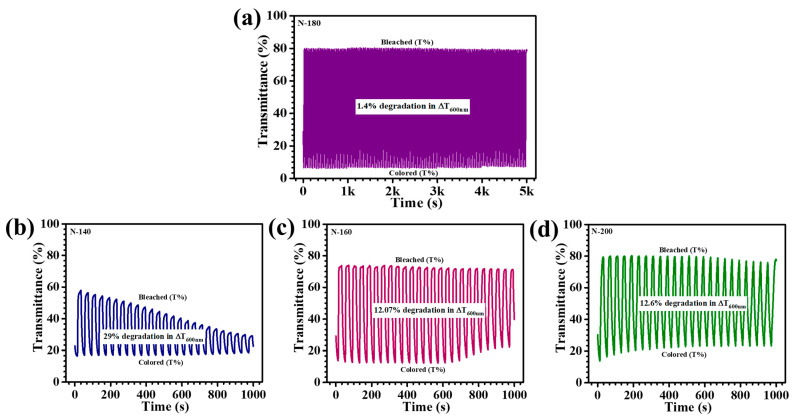
(**a**) The long-term transmittance stability over 5000 s duration of N-180 sample, transmittance stability profiles of (**b**) N-140, (**c**) N-160, and (**d**) N-200 samples measured for 1000 s.

The long-term cycling stability of EC materials plays a pivotal role in determining their viability for smart window applications, where durability and consistent performance over extended cycles are paramount. In this study, the influence of hydrothermal reaction temperature on the cycling stability of Nb_2_O_5_ thin films was systematically examined through in-situ transmittance measurements at 600 nm during repeated coloration and bleaching cycles. The films were subjected to alternating potentials within a three-electrode electrochemical setup, within a voltage range of +1 V to −2 V. Among the synthesized films, the N-180 sample exhibited superior EC stability, retaining a remarkable optical modulation with an almost negligible decline of 1.4% over 5000 s of continuous cycling (Figure 10a). The film demonstrated sustained EC reversibility, maintaining a stable coloration and bleaching response even after prolonged ion intercalation/deintercalation cycles, showcasing a consistent EC response without significant attenuation, thereby confirming the film’s robust electrochemical durability. In contrast, the other samples, N-140, N-160, and N-200 displayed a considerable decline in their EC performance over 1000 s of cycling, with optical modulation reductions of 29%, 12.07%, and 12.6%, respectively (Figure 10b N-140, Figure 10c N-160, and Figure 10d N-200). The pronounced degradation observed in these films can be ascribed to the presence of excess ion-trapping sites and structural inconsistencies, which hinder efficient charge transfer and induce irreversible electrochemical losses. Over time, N-140 film exhibited a noticeable decrease in their bleached and colored state transmittance, while for N-160 and N-200 films, their colored-state transmittance relatively unstable, indicative of ion accumulation and limited reversibility in charge exchange processes. The exceptional stability of the N-180 film resulted from its optimal nanostructural configuration, achieved through precise hydrothermal temperature modulation. The film’s uniform morphology and well-defined porous network enhance Li^+^ ion diffusion dynamics, mitigating ion-trapping effects and ensuring sustained EC activity. Thus, by fine-tuning the synthesis temperature, a significant enhancement in EC material longevity and performance can be achieved, paving the way for next-generation energy-efficient smart window technologies.

**Figure 11:**
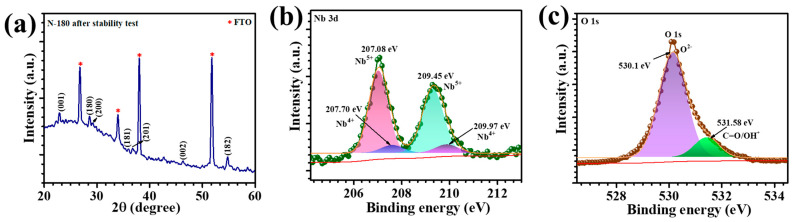
(**a**) XRD pattern of N-180 sample after stability test, (**b**) Nb 3d core-level spectrum, and (**c**) O 1s core-level spectrum of N-180 film after stability.

The XRD pattern of the N-180 thin film after long-term cycling is presented in Figure 11a. The diffraction peaks remain well-aligned with those of the pristine sample, indexed to the T-Nb_2_O_5_ phase (JCPDS card no. 00-030-0875). This suggests that the overall crystal structure remains stable even after extended electrochemical cycling. However, a slight shift in peak positions and minor noise in the XRD pattern indicate a small degree of structural distortion, possibly due to lattice strain or minor phase relaxation caused by prolonged ion intercalation and deintercalation during cycling. To further investigate degradation at the electronic structure level, the XPS analysis was conducted on the N-180 thin film post-cycling (Figure 11b,c). The high-resolution Nb 3d spectrum (Figure 11b) exhibit the characteristic Nb^5+^ peaks at 207.08 eV and 209.45 eV, consistent with the pristine sample. However, additional peaks at 207.70 eV and 209.97 eV corresponding to the Nb^4+^ state are observed after cycling. The emergence of Nb^4+^ states suggests partial reduction of Nb^5+^ due to prolonged redox cycling, indicating a gradual loss of oxygen or subtle changes in the local Nb-O bonding environment. In contrast, the O 1s spectrum (Figure 11c) exhibits no significant new features apart from a slight peak shift, suggesting minor structural rearrangements rather than severe degradation. Based on the XRD and XPS findings, the primary degradation mechanism appears to involve subtle lattice distortions and partial Nb^5+^ to Nb^4+^ reduction rather than drastic structural collapse or severe electrolyte-induced degradation. These observations suggest that while the N-180 thin film maintains its crystalline integrity over extended cycling, minor structural variations and electronic state changes occur, which could impact long-term performance.

Over the past 25 years, extensive research has been conducted on pristine and doped Nb_2_O_5_ thin films for EC applications. However, comparing these studies remains challenging due to the varying synthesis conditions and processing techniques. To address this, we have compiled a comparative analysis of Nb_2_O_5_-based EC materials, highlighting the superior EC performance of our optimized N-180 sample, presented in Table 2. The T-Nb_2_O_5_ phase with a well-defined nanostructured morphology is a key factor contributing to its exceptional coloration efficiency, optical modulation, and electrochemical reversibility. Several recent studies have explored Nb_2_O_5_-based heterostructures and composite systems to enhance EC properties including Y. Park et al. reported WO_3_/Nb_2_O_5_ stacked films for electrochromic application by sputtering method. They found irregular shaped particles morphology having 82.21% optical modulation and 83.33 cm^2^/C coloration efficiency [32]. E. Costa et al. studied Nb_2_O_5_-TiO_2_ mixed oxides, synthesized via sol-gel/dip coating method. The shown homogeneous small particles exhibited 60% optical modulation [33]. Furthermore, A. Santhosh et al. reported the study of Nb_2_O_5_-NiO mixed oxide films prepared by simple spin coating technique, which absorbed 65% IR radiation [34]. N. Usha et al. reported the electrochromic study of Nb_2_O_5_:MoO_3_ thin films prepared by RF magnetron sputtering. This study exhibited the coloration efficiency of 2.303 mm^2^/C [35]. N. Akkurt et al. studied graphene doped Nb_2_O_5_ thin films prepared by a thermionic vacuum arc technique. They reported the highest coloration efficiency of 56 cm^2^/C at 550 nm with optical modulation of 42% [36]. C. Tang et al. studied WO_3_-Nb_2_O_5_ electrochromic films deposited via magnetron sputtering. The film exhibited larger grains surface morphology that showed 75.5% transmittance modulation at 633 nm with 35.2 cm^2^/C coloration efficiency [37]. G. Azevedo et al. reported sol-gel prepared Nb_2_O_5_:Li^+^:V_2_O_5_ material for advanced EC application; it demonstrated 66% of optical transmittance at 633 nm [38]. A. Kumar et al. studied W-doped Nb_2_O_5_, synthesized via dip coating method exhibited nano-fibrous morphology. They demonstrated 68.7 cm^2^/C coloration efficiency at 600 nm [39]. By integrating these insights, we demonstrate that the N-180 Nb_2_O_5_ thin film outperforms several reported systems, particularly in terms of coloration efficiency, reversibility, and overall EC response. The exceptional performance is attributed to the T-Nb_2_O_5_ crystal structure, which provides enhanced ion transport pathways and a stable nanostructured morphology. This reinforces the novelty and scalability of our approach for practical EC applications.

## 5. Electrochromic Device

**Figure 12:**
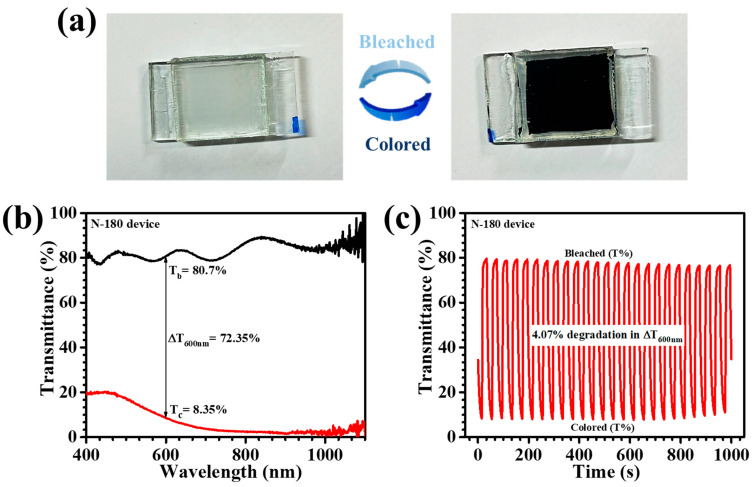
(**a**) Photograph of N-180 EC device in its colored and bleached states, (**b**) in-situ transmittance spectrum of N-180 device, and (**c**) stability test of the EC device.

The practical realization of EC materials into functional devices is a fundamental step toward their commercial integration, particularly for advanced smart window technologies. In this study, the superior-performing N-180 thin film, synthesized via a hydrothermal approach, was employed as the core EC component in a large-scale prototype device. The architectural configuration adhered to a conventional sandwich-type assembly, encompassing an EC working electrode, an electrolyte medium, and a counter electrode layer, ensuring optimal charge transport and electrochemical stability. The fabricated device exhibited exceptional EC modulation, as evidenced by the distinct optical transition captured in Figure 12a. Upon the application of an external voltage, the device transitioned seamlessly from a transparent (bleached) state to a deep blue (colored) state, underscoring its dynamic EC functionality. To quantify its EC dynamics, in-situ transmittance spectra were recorded over the 400–1000 nm wavelength range (Figure 12b), revealing a substantial optical modulation, peaking at 72.35% at 600 nm. Such a remarkable performance affirms the superior light regulation capability of the synthesized Nb_2_O_5_ film, aligning with the demands for energy-efficient smart windows and display technologies. This striking color shift endorses the excellent Li^+^ ion intercalation/deintercalation capability of the N-180 thin film, which is essential for achieving high contrast and rapid switching kinetics. Furthermore, the long-term durability of the EC device was examined through cyclic coloration and bleaching over a period of 1000 s (Figure 12c). The optical contrast remained stable during the initial cycles, confirming the structural integrity of the material under continuous operation. However, a slight decline of 4.07% in optical modulation was observed over prolonged cycling. Despite this minor degradation, the overall stability of the EC response validate the scalability of the synthesized films. The results unequivocally demonstrate that the hydrothermal temperature-optimized N-180 thin film not only delivers superior EC performance on electrode scale but also retains its efficacy in large-scale device configurations. This breakthrough strengthens its potential for next-generation smart windows and other advanced optoelectronic applications, where efficiency, durability, and scalability are paramount.

## 6. Conclusions

In summary, this study precisely investigated the influence of hydrothermal temperature variation on the structural, morphological, and EC properties of Nb_2_O_5_ thin films, shedding light on their potential for next-generation energy-efficient smart window applications. The controlled synthesis of Nb_2_O_5_ at different temperatures (140 °C, 160 °C, 180 °C, and 200 °C) revealed a profound impact on surface morphology, crystallinity, and electrochemical behavior. At optimal temperature (180 °C), a well-defined nanostructured morphology emerged, characterized by a highly porous framework with interconnected grains. This structural evolution enhanced the electrochemical active surface area, facilitating efficient charge transfer and superior ion intercalation/deintercalation kinetics, thereby maximizing the material’s EC capabilities. Notably, the N-180 film demonstrated the most favorable EC performance, exhibiting a pronounced optical modulation of 74.13% at 600 nm, rapid switching dynamics (t_c_ = 12.5 s and t_b_ = 5.6 s) and high coloration efficiency of 81.33 cm^2^/C. Moreover, cyclic stability assessments revealed that the film retained a considerable fraction of its EC activity over prolonged operational cycles, affirming the structural robustness of the synthesized material. The assembled EC device, incorporating the optimized thin film, showcased excellent reversible optical transitions, with a substantial modulation in the visible region. The insights gained from this study provide a strong foundation for advancing Nb_2_O_5_-based EC materials, paving the way for their integration into smart energy devices, flexible optoelectronics, and adaptive display technologies. Ultimately, this work contributes significantly to the growing demand for high-performance, scalable EC materials, fostering innovations in sustainable energy and intelligent optical systems.

## Figures and Tables

**Table 1:** Electrochromic performance evaluation of N-140, N-160, N-180, and N-200 thin films synthesized at variable hydrothermal temperatures.

Sample Code	Charge Intercalation (Q_i_) (C/cm^2^)	Charge Deintercalation (Q_di_) (C/cm^2^)	Reversibility (%)	Coloration Time (s) (t_C_)	Bleaching Time (s) (t_b_)	T_b_ %	T_C_ %	Optical Modulation (ΔT_600nm_%)	Optical Density (ΔOD)	Coloration Efficiency (cm^2^/C)
**N-140**	0.058	0.056	97%	13.2	5.3	58.7%	16.07	42.63%	2.5	43.10
**N-160**	0.065	0.064	98%	12.8	4.6	74.05%	13.41%	60.64%	3.4	52.3
**N-180**	0.06	0.0598	99%	12.5	5.6	81.2%	7.07%	74.13%	4.88	81.33
**N-200**	0.068	0.066	98%	14.1	9.5	80.02%	13.2%	66.82%	3.6	53.04

**Table 2:** Comparative analysis of electrochromic performance with reported Nb_2_O_5_-based compositions.

Sr. No.	Material	Method	Morphology	ΔT%	Coloration Efficiency (cm^2^/C)	Switching Speed	Device ΔT%	Stability	Ref.
1	WO_3_/Nb_2_O_5_	Sputtering	Irregular particles	82.21 (630 nm)	83.33	t_b_: 0.55 s t_c_: 1.79 s	-	3500 cycles (40% loss)	[32]
2	Nb_2_O_5_-TiO_2_	Sol-gel/dip coating	Small particles	60%	-	-	-	200 s	[33]
3	Nb_2_O_5_-NiO	Spin coating	-	65% IR	-	-	-	-	[34]
4	Nb_2_O_5_:MoO_3_	Sputtering	-	20% (630 nm)	2.303 mm^2^/C	-	-	-	[35]
5	Graphene-doped Nb_2_O_5_	Thermionic vacuum arc	-	42% (550 nm)	56	t_b_: 3.4 s t_c_: 1.5 s	-	2000 s	[36]
6	WO_3_-Nb_2_O_5_	Sputtering	Larger grain	75.5% (633 nm)	35.2	t_b_: 0.7 s t_c_: 7 s	33%	180 cycle	[37]
7	Nb_2_O_5_:Li^+^:V_2_O_5_	Sol-gel	Small grains	66% (633 nm)	-	-	-	-	[38]
8	W-doped Nb_2_O_5_	Dip coating	Nano-fibrous	-	68.7 (600 nm)	t_b_: 3.5 s t_c_: 25.3 s	-	-	[39]
9	N-180	Hydrothermal	Nanospheres	74.13% (600 nm)	81.33	t_b_: 5.6 s t_c_: 12.5 s	72.35%	5000 s	This work

## Data Availability

The data presented in this study are available on request from the corresponding author. The data are not publicly available due to restrictions.
